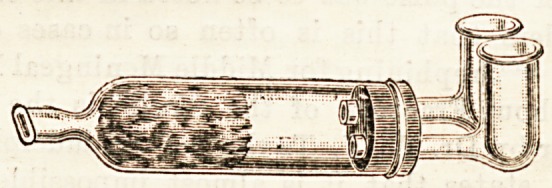# New Appliances and Things Medical

**Published:** 1895-07-13

**Authors:** 


### NEW APPLIANCES AND THINGS MEDICAL.

[We shall be glad to receive, at our Office, 428, Strand, London, W.C., from the manufacturers, specimens of all new preparations and appliances which may be brought out from time to time.]

### VITALIA MEAT JUICE.

The Vitalia Meat Juice Company, of Waltham Buildings, 1, Holborn Circus, have sent us samples of their manufacture for notice. Vitalia Meat Juice is a viscid port-wine coloured fluid, with but little odour and a bland sweetish taste. It contains a very large amount of coagulable albumen. The paper sent with the sample states more than 20 per cent.; our analysis calculated from the nitrogen of the meat extract found proves this to be true, as it was found to be equal to 20·25 per cent. If heated in a test tube the fluid becomes solid, and alcohol in excess also causes a dense precipitate. The salts of iron and phosphorus are also present in considerable quantity, the latter, calculated as phosporic acid, being present in nearly 1 per cent. of the fluid. The albumen is mainly in such a form as to be readily absorbed. Microscopically altered blood cells, small fat globules, and a little granular detritus are to be seen. The juice is free from such preservatives as salicylic and boracic acids; five per cent. of chloride of sodium and a minute trace of another non-objectionable substance being the sterilising agents employed. Our examination proves this to be a very concentrated albuminous fluid of high food value. The next questions are: Can it be easily administered, and does the patient like it? We have tried it in a case of chronic septic peritonitis (three months' duration), when the temperature rose and there was sickness. Diluted with soda-water, or given with a little weak lemonade, it acted well, and not only was taken without repugnance but also without nausea or sickness following. Also in several cases of infantile diarrhœa well diluted during the present hot weather. Here it answered admirably. Also in a case of the vomiting of pregnancy with exhaustion, both by the mouth and by the rectum; it was well retained; very small doses were used by the mouth, 30 drops at two hours' intervals. In conclusion, we consider this to be a very useful preparation. Not only does the analysis prove its food strength, but clinical observation bears this out in showing its sustaining value.

### STAINER'S CHLORIDE OF AMMONIUM INHALER.

(Wm. Toogood, Heddon Street, Regent Street, W.)

This is a very simple and efficient form of chloride of ammonium inhaler. Two plugs of asbestos are saturated with ammonia and hydrochloric acid solutions respectively.

The patient by inspiration draws the air through these plugs which, becoming impregnated from each of them, is mixed in a larger tube, and the resulting chloride of ammonium vapour inhaled. A damp sponge is arranged so as to prevent any excess of ammonia reaching the mouth. The apparatus is altogether very convenient, and cannot possibly get out of order.

## Figures and Tables

**Figure f1:**